# Plasma Neurofilament Light Chain Predicts Mortality and Long-Term Neurological Outcomes in Patients with Intracerebral Hemorrhage

**DOI:** 10.14336/AD.2022.21020

**Published:** 2023-04-01

**Authors:** Pei Zheng, Xuejiao Wang, Jingshan Chen, Xinli Wang, Samuel X Shi, Kaibin Shi

**Affiliations:** ^1^Department of Neurology, National Clinical Research Center for Neurological Diseases of China, Beijing Tiantan Hospital, Capital Medical University, Beijing 100070, China.; ^2^Center for Neurological Diseases, The Third People’s Hospital of Datong, Datong 037046, China.; ^3^Department of Neurology, Institute of Neuroimmunology, Tianjin Medical University General Hospital, Tianjin 300052, China.; ^4^Clinical Neuroscience Research Center, Departments of Neurosurgery and Neurology, Tulane University School of Medicine, New Orleans, LA 70122, USA.

**Keywords:** neurofilament light chain, intracerebral hemorrhage, long-term outcome

## Abstract

Patients with intracerebral hemorrhage (ICH) often suffer from heterogeneous long-term neurological deficits, such as cognitive decline. Our ability to measure secondary brain injury to predict the long-term outcomes of these patients is limited. We investigated whether the blood neurofilament light chain (NfL) can monitor brain injury and predict long-term outcomes in patients with ICH. We enrolled 300 patients with first-episode ICH within 24 h recruited in the Chinese Cerebral Hemorrhage Mechanisms and Intervention study cohort from January 2019 to June 2020. Patients were prospectively followed up for 12 months. Blood samples were collected from 153 healthy participants. Plasma NfL levels determined using a single-molecule array revealed a biphasic increase in plasma NfL in ICH patients compared to healthy controls, with the first peak at around 24 h and a second elevation from day 7 through day 14 post-ICH. Plasma NfL levels were positively correlated with hemorrhage volume, National Institute of Health Stroke Scale, and Glasgow Coma Scale scores of ICH patients. Higher NfL concentration within 72 h after ictus was independently associated with 6- and 12-month worsened functional outcomes (modified Rankin Scale ≥ 3) and higher all-cause mortality. Magnetic resonance imaging and cognitive function evaluation were available for 26 patients at 6 months post-ICH, and NfL levels measured 7 days post-ictus correlated with decreased white matter fiber integrity and poor cognitive function at 6 months after stroke. These findings suggest that blood NfL is a sensitive marker for monitoring axonal injury post-ICH and can predict long-term functional ability and survival.

## Introduction

Intracerebral hemorrhage (ICH) accounts for 10% to 20% of all strokes and is the most devastating subtype of stroke, with high disability and mortality rates [1-3]. Survivors of ICH are at high risk of developing varying degrees of neurological, physical, and cognitive impairment [4]. Although structural damage by the hematoma directly causes acute symptoms, long-term neurological deficits are not prominently associated with acute ICH characteristics [5, 6], suggesting potentially different mechanisms underlying long-term neural damage. The severity of acute ICH can be well evaluated using clinical scales such as the ICH score (admission GCS score, initial hematoma volume, presence of intraventricular hemorrhage, infratentorial ICH origin, and age), which is also associated with mortality at 30 days and even long-term functional outcomes [7]. Additionally, bleeding lesion characteristics can now be thoroughly described by neuroimaging approaches, including MRI and CT scans, and established imaging markers such as perihematomal edema and white matter integrity can partially assist in predicting neurological outcomes in ICH patients [8-10]. Furthermore, recent studies have identified a group of blood biomarkers that are reportedly associated with long-term outcomes of ICH, including but not limited to the neutrophil-to-lymphocyte ratio, complement component C1q, interleukin-6, and stress hyperglycemia [11-13]. However, while these markers play critical roles in characterizing hemorrhage features and are valuable predictors of ICH outcomes, they have not been directly validated for neuronal injury. Therefore, continuous monitoring of neuroaxonal injury remains a critical yet unmet need as it can help understand and predict long-term prognosis in these patients [14].

The neurofilament light chain (NfL) is a major cytoskeletal component of neuronal axons and is abundantly and exclusively expressed in neurons. NfL is released into the extracellular fluid during acute and chronic axonal injury and is associated with the severity of the injury [15]. Recently, the development of a single-molecule detection array (Simoa) has made it possible to detect a relatively low level of NfL in the blood, which reliably reflects NfL levels in the cerebral spinal fluid and brain interstitial fluid. Plasma NfL has been studied as a promising candidate biomarker for neuronal injury and has shown prognostic value in several neurological disorders, including multiple sclerosis, as well as in neurodegenerative disorders, such as Alzheimer’s and Parkinson’s disease [16-18]. In acute brain injuries, such as acute cerebral infarction [19], cerebral amyloid angiopathy-related intracerebral hemorrhage [20], aneurysmal subarachnoid hemorrhage [21, 22], and traumatic brain injury [23, 24], plasma NfL levels are positively associated with the severity of brain injury. Two studies that recruited 37 and 29 ICH patients reported that plasma NfL quantified within three days post-ictus was independently associated with functional disability and mortality in ICH patients [25, 26]. However, the conclusions from these two studies were limited by their small cohort sizes and relatively short follow-up times. Furthermore, the temporal dynamics of plasma NfL post-ICH and its predictive value for long-term outcomes remain unclear. The present study assessed plasma NfL changes from acute to subacute phases in a relatively large ICH population to evaluate the prognostic value of NfL levels on mortality, post-ICH functional disability, long-term neurological deficits of ICH patients, and its association with chronic neuroanatomical changes via neuroimaging.

## MATERIALS AND METHODS

### Study populations

This study analyzed data from the Chinese Cerebral Hemorrhage Mechanisms and Intervention study cohort (ID:2018YFC1312200), which recruited 311 patients admitted to the Department of Neurosurgery and Neurology at Datong Third Hospital from January 2019 to June 2020. Patients aged > 18 years, with first-episode ICH within or at 24 h were included. ICH was diagnosed following the Guidelines for Diagnosis and Treatment of Cerebral Hemorrhage (2014). The study exclusion criteria were primary subarachnoid hemorrhage, hemorrhage transformation of cerebral infarction, hemorrhage after thrombolysis, traumatic cerebral hemorrhage, subdural/extradural hematoma, other diseases, neurodegenerative diseases, tumors, cognitive impairment, and dementia. Overall, 311 patients were recruited. Medical data was unavailable for three patients, and blood samples were unavailable for eight others. Plasma from 153 healthy subjects was obtained from the medical examination centers of the Beijing Tiantan Hospital and Tianjin Medical University General Hospital. None of the study participants had a prior history of neuropsychiatric disease, stroke, dementia, or underlying diseases at the time of blood sampling. The study was conducted in accordance with the World Medical Association Declaration of Helsinki and approved by the local ethics committee (No: S485). Written informed consent was obtained from the patient or a legally responsible relative.

At recruitment, patients were scored using the Glasgow Coma Scale (GCS) and National Institute of Health Stroke Scale (NIHSS) at admission and at the time of each blood collection. Diabetes was defined as a specified diagnosis in the medical chart or accompanying pharmacological anti-diabetic treatment. Hypertension was defined as a history of antihypertensive treatment before hospital admission. Smoking and alcohol abuse were indicated in the medical chart. A neuroradiology consultant evaluated computed tomography (CT) scans, and ICH volume was quantified using the ABC/2 method. Patients were regularly followed up to evaluate functional outcomes using the modified Rankin Scale (mRS) at 1, 3, 6, and 12 months via phone calls or in-person interviews up to June 2021 or patient’s death. Additionally, 26 patients admitted to the hospital 6 months post-ICH were available for cognitive function evaluation using Montreal cognitive assessment (MoCA) and magnetic resonance imaging. The characteristics of subjects included in this study are provided in Table 1.

**Table 1:** Characteristics of controls and ICH patients.

Variable, No. (%)	Controls (n=153)	All ICH patients (n=300)		Patients with blood samples available at days 7 and 14 (n=41)		Patients received MoCA and MRI evaluation at 6 months (n=26)	
Age, years (median (minimum, maximum))	46 (25, 79)	59 (27, 84)		63 (35, 77)		63 (35, 77)	
Male	73 (48.7)	204 (68.0)		34 (82.9)		22 (84.6)	
Medical history							
Diabetes,	N/A	34 (11.3)		0 (0)		0 (0)	
Hypertension,	N/A	197 (65.7)		37 (90.2)		22 (84.6)	
Cerebrovascular Disease	N/A	35 (11.7)		2 (4.8)		2 (7.7)	
Ischemic Heart Disease	N/A	10 (3.3)		0 (0)		0 (0)	
Cancer	N/A	4 (1.3)		0 (0)		0 (0)	
Current smoking status	N/A	117 (39.0)		16 (39.0)		9 (34.6)	
Clinical evaluations at admission							
Glasgow Coma Scale							
15-14	N/A	132 (44.0)		19 (46.3)		13 (50.0)	
13-9	N/A	133 (44.3)		18 (43.9)		10 (38.5)	
8-3	N/A	35 (11.7)		4 (9.7)		3 (11.5)	
NIHSS							
0-4	N/A	45 (15.0)		3 (7.3)		3 (11.5)	
5-15	N/A	183 (61.0)		21 (51.2)		14 (53.8)	
16-20	N/A	14 (4.7)		17 (41.5)		9 (34.6)	
21-42	N/A	44 (14.7)					
Hemorrhagic features							
Location of hemorrhage							
Hemisphere	N/A	47 (15.7)		8 (19.5)		5 (19.2)	
Basal ganglia	N/A	168 (56.0)		21 (51.2)		12 (46.1)	
Thalamus	N/A	26 (8.7)		8 (19.5)		6 (23.1)	
Brain stem	N/A	16 (5.3)		3 (7.3)		2 (7.7)	
Cerebellum	N/A	34 (11.7)		3 (7.3)		2 (7.7)	
Hemorrhage volume, Median (IQR), mL	N/A	15 (26)		15 (23)		13 (23)	
Intraventricular bleeding	N/A	82 (27.3)		16 (39.0)		10 (38.5)	
Surgical treatment	N/A	128 (42. 7)		20 (48.8)		11 (42.3)	
Outcomes							
Death	N/A	69 (23)		3 (7.3)		0 (0)	
Modified Rankin Scale score	N/A	mRS < 3	mRS≥3	mRS < 3	mRS≥3	mRS < 3	mRS≥3
1 month	N/A	59 (22.6)	202 (77.4)	7 (17.1)	32 (78.0)	4 (15.4)	22 (84.6)
3 months	N/A	105 (44.9)	129 (55.1)	14 (34.1)	25 (60.9)	10 (38.5)	16 (61.5)
6 months	N/A	151 (65.1)	81 (34.9)	26 (63.4)	12 (29.2)	20 (76.9)	6 (23.1)
12 months	N/A	173 (74.9)	58 (25.1)	29 (70.7)	9 (21.9)	22 (84.6)	4 (15.4)

Data are presented as numbers (%) or as indicated. NIHSS, National Institutes of Health Stroke Scale; IQR, interquartile range; N/A, not available.

### Blood sampling and NfL measurement

Peripheral blood samples were collected into ethylenediaminetetraacetic acid (EDTA) tubes from patients with ICH within 72 h of the episode. Blood samples at days 7 and 14 post-ICH were available for 41 patients without a predefined screening plan, and the characteristics of these patients are provided in Table 1. Samples were processed by centrifuging at 3000*g* for 5 min, and plasma was stored at -80 °C. Plasma NfL levels were measured using single-molecular array (Simoa) technology with an ultra-high sensitivity protein molecular detection instrument (Simoa HD-1, Quanterix Corporation, MA, USA) and Simoa NfL reagent kit (502153, Quanterix Corporation). The limit of detection (LoD) for NfL was 0.038 pg/ml. Samples were analyzed in two analytical sessions using the NfL kit with the same lot number.

### MRI protocols and imaging analysis

Twenty-six patients were available for MRI scanning six months after ICH. All MRI data were acquired using a 3.0-Tesla GE MR scanner (SIGNA Pioneer, General Electric, Milwaukee, WI, USA) at the Third People’s Hospital of Datong. Brain MRI sequences including axial 3-dimensional (3D) T1-weighted images (TR/TE = 8.3/3.2 ms, flip angle=12°, FOV=240 mm×240 mm, slice thickness=1 mm, gap=0, slice number=156), T2-weighted fluid-attenuated inversion recovery (FLAIR) (TR/TE/TI=8000/120/2100 ms, FOV=240 mm×240 mm, slice thickness=5.0 mm, gap= 1 mm, slice number = 23), and diffusion tension imaging (DTI, TR/TE=8000/77 ms; FOV=240 mm×240 mm; slice thickness=5 mm with no gap; 32 encoding diffusion directions with one values of b [b=1000] for each direction; and 10 non-diffusion-weighted images [b=0 s/mm^2^], slice number=30).

White matter lesions were labeled by an experienced neuroradiologist from T2- FLAIR images using a 3D-slicer (www.slicer.org/). DTI data preprocessing was performed using the FMRIB’s diffusion toolbox (FDT, http://www.fmrib.ox.ac.uk/fsl, FSL 4.0). First, eddy current distortions and motion artifacts were corrected using the affine alignment of each diffusion-weighted image to the image of b = 0 s/mm^2^. Subsequently, FMRIB’s Brain Extraction Toolbox (BET) was used to remove non-brain tissue from the average b0 image, and a brain mask was applied to the rest of the diffusion-weighted images. Third, the diffusion tensor was estimated for each voxel using the DTIFIT function via linear regression to derive FA maps. Fourth, the FA map was registered to MNI space using FLIRT linear registration. Normalized, modulated, and smoothed white matter density maps were used as masks to extract the FA values of these patients for further analysis. Gray matter volume was evaluated using voxel-based morphometry (VBM) analysis performed using the CAT12 Toolbox (http://www.neuro.uni-jena.de/cat) implemented in the statistical parameter mapping software (SPM12, http://www.fl.ion.ucl.ac.uk/spm). T1 images were normalized to template space and segmented into gray matter, white matter, and cerebrospinal fluid. After spatial preprocessing, normalized, modulated, and smoothed gray matter density maps were used for the statistical analysis (Supplementary Figure 1).

### Statistical Methods

For all analyses, the significance level was set at 0.05, and statistical analyses were performed using IBM SPSS version 19.0 (IBM Corp., Armonk, N.Y., USA). The normality of data distribution was assessed using the Shapiro-Wilk test. Continuous variables are described as mean±standard deviation or median with interquartile range, and categorical variables are presented as numbers and percentages. After adjusting for age, the Mann-Whitney U test was used to compare plasma NfL levels between ICH patients and healthy controls. NfL concentrations were examined on the base 2 logarithmic scale in all analyses of the validation series because of their skewed distribution. In patients with ICH (overall and stratified according to hemorrhage location, ventricular extension, surgery), associations of NfL with hemorrhage volume at admission (ABC/2), measures obtained at the time of blood collection (NIHSS and GCS), and post-blood collection outcomes (1-, 3-, 6-, 12-month mRS and survival) were evaluated using regression models appropriate for the nature of the given outcome measures. Specifically, associations between NfL and ABC/2, NIHSS, and GCS were examined using linear regression models, where ABC/2, NIHSS, and GCS were examined on the square root scale because of their skewed distribution. β coefficients and 95% confidence intervals (CIs) were estimated and interpreted as the change in mean ABC/2, NIHSS, and GCS scores (on the square root scale) for each fold of the NfL concentration. Associations between NfL and mRS scores were evaluated using binary logistic regression models, while mRS was dichotomized as < 3 or ≥ 3. Odds ratios (ORs) and 95% CIs were estimated. Finally, associations between NfL concentration and survival were evaluated using Cox proportional hazards regression models. The baseline time point for survival was set as the date of blood collection, and censoring was performed on the date of the last follow-up; hazard ratios (HRs) and 95% CIs were estimated. All HRs and ORs estimated corresponded to a doubling in the NfL concentration. All regression models (Cox, linear, logistic) were initially adjusted for the time from ICH to blood collection and subsequently for age, sex, smoking, hypertension, history of cerebrovascular disease, and diabetes. We further evaluated the ability of NfL to independently predict 6- and 12-month mRS scores and survival at 12 months using Cox regression models. When evaluating survival at 12 months, we calculated the area under the receiver operating characteristic (ROC) curve (AUC). The optimal cut-off point was chosen as the NfL value with the maximum correct classification, and the positive/negative likelihood ratio for that point was calculated. We also analyzed the Kaplan-Meier curve with the log-rank test of predictors above and below the cut-off point. When examining the 6-month and 12-month mRS (differentiated as <3 or ≥ 3), AUC was estimated both with and without NfL in the multivariable model. The associations between NfL concentrations, image markers, and cognitive function were evaluated using Spearman’s rank correlation test.

## RESULTS

### Plasma NfL increased in a two-phase mode post intracerebral hemorrhage

**Figure 1.**
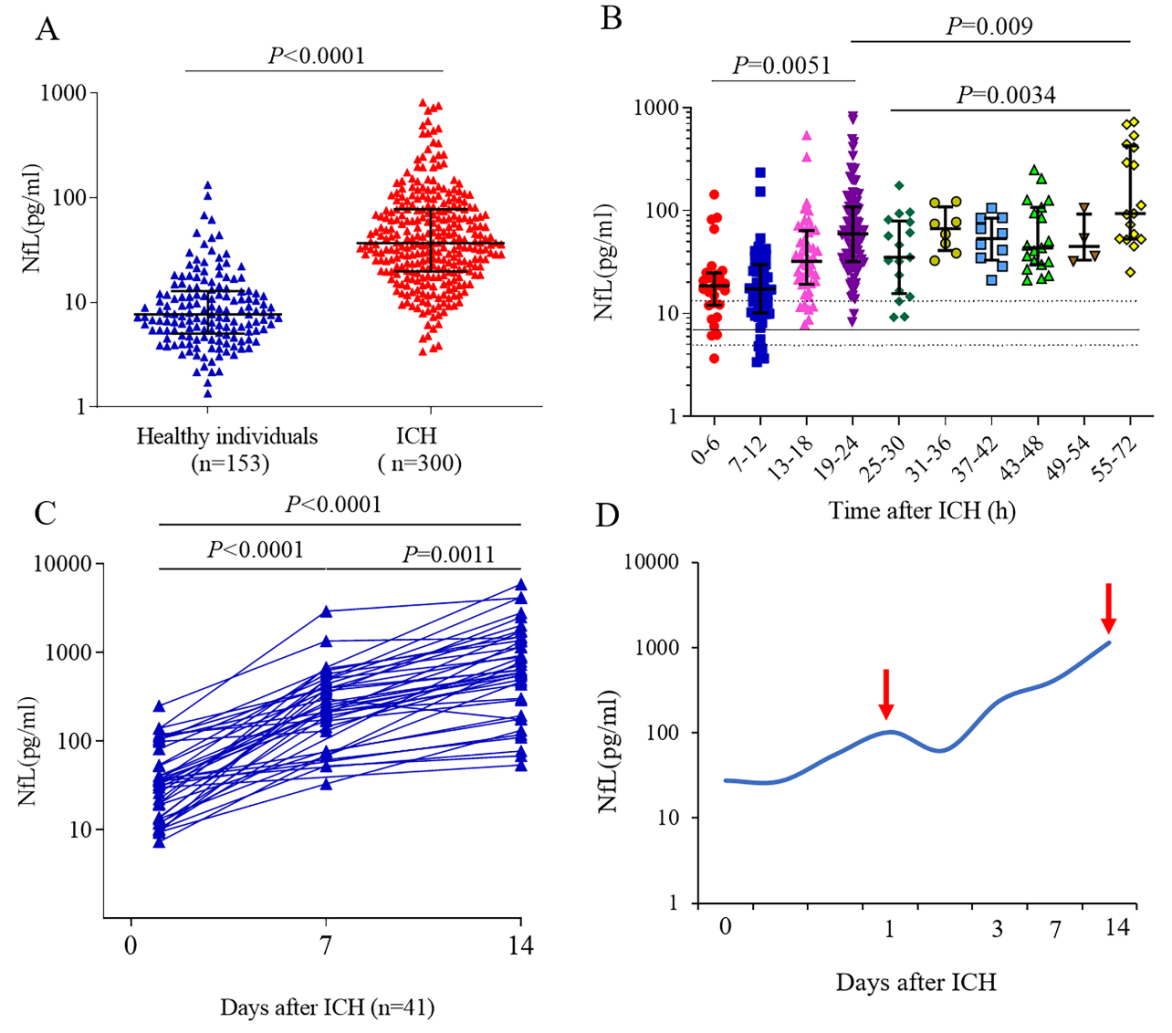
Plasma neurofilament light chain is elevated bimodally following ICH in patients. (A) Comparison of plasma NfL levels of ICH patients within 72 hours of ictus with those of healthy individuals, adjusted by age. Individual patient data are shown. Lines indicate the median with the interquartile range. (B) NfL concentrations based on timing from ICH to blood collection (0 to 72 hours). The median plasma NfL concentration of controls (n = 153) is depicted as a solid horizontal line; horizontal dotted lines denote the interquartile range. (C) Blood samples were collected in 41 ICH patients within 24 hours, at day 7, and day 14; continuous change in plasma NfL at indicated time points were evaluated in these patients. (D) The trend curve of plasma NfL changes after ICH; red arrows indicate two peaks at 24 hours and 14 days post-ICH. NfL, neurofilament light chain; ICH, intracerebral hemorrhage. Data are shown as the median±interquartile range, and the Y axis of NfL concentration are shown on the base 10 logarithmic scale. The statistical significance of the differences was calculated using the Mann-Whitney U test. Controls, n=153; ICH patients, n=300. P values are shown in the figure.

The demographic and clinical characteristics of patients with ICH are presented in Table 1. After excluding participants with incomplete data, 300 patients with ICH with an average age of 59 years (range, 27-84 years) were included in the analysis. Compared to healthy controls (12.3±1.3 pg/mL), plasma NfL was significantly elevated in patients with ICH (79.8±8.4 pg/mL, *P* < 0.001, adjusted by age; Fig. 1A) within 72 hours of disease onset, consistent with two prior reports included smaller ICH populations [25, 26]. Similar to findings reported in healthy subjects [27], plasma NfL of ICH patients was correlated with age but was comparable between female and male patients (Supplementary Fig. 2). We then classified the patients according to the time from symptom onset to blood collection, which enabled us to trace the plasma NfL changes at 6-hour increments from disease onset to day 3 post-ictus. We observed a continuous increase in plasma NfL after ICH onset, peaking approximately 24 h after ictus (Fig. 1B). To further characterize the change in NfL from the acute to subacute phases, we repeatedly measured plasma NfL levels at days 7 and 14 in 41 patients. Interestingly, NfL levels at day 7 post-ICH were significantly higher than those measured within 24 h and continuously increased up to day 14 (Fig. 1C). These data characterized the dynamic kinetics of plasma NfL, changing in two phases after ICH: a primary increase within 24 h of ICH, and a secondary increase during the subacute phase, suggesting two stages of neuroaxonal injury following ICH (Fig. 1D).

### Plasma NfL correlates with the disease severity of intracerebral hemorrhage in the acute phase

To determine the relationship between NfL and acute brain tissue damage, we evaluated the correlation between plasma NfL levels and hemorrhagic volume in the brain using the ABC/2 formula (Table 2). When adjusting for the time from hemorrhagic episode to blood collection, NfL levels were positively associated with hemorrhage volume (*P* < 0.001), which remained significant when further adjusted for the following potential confounding factors: time from hemorrhage to blood collection, age, sex, smoking, hypertension, cerebrovascular disease, and diabetes (*P* < 0.001). We subsequently determined whether hemorrhage location, ventricle extension, and surgical treatment affected NfL levels. In our stratified analyses, NfL levels were significantly associated with ABC/2 values in patients with supratentorial hemorrhage (*P* < 0.001) after adjusting for the time from hemorrhage to blood collection and in the remaining patients after further adjusting for confounding factors (*P* = 0.001) (Table 2). In patients with infratentorial hemorrhage, the correlation between NfL and hemorrhage volume was no longer significant (Table 2). Similarly, the association between NfL and hemorrhagic volume was significant only in patients with parenchymal hemorrhage after adjustment (*P* = 0.001), while in patients with hemorrhage extending to the ventricles, the correlation between NfL and hemorrhage volume was no longer significant (Table 2). In patients who did not receive surgical treatment for hematoma removal, the correlation between NfL and ABC/2 was weaker than in those who underwent surgery (Table 2). Notably, the NfL levels on days 7 and 14 were no longer associated with ABC/2 at admission (Supplemntary Table 1).

**Table 2:** Association of NfL concentrations with ABC/2 at blood collection.

		Adjusting for the time from hemorrhage to blood collection		Adjusting for the time from hemorrhage to blood collection, age at blood collection, sex, current smoking, hypertension, cerebrovascular disease, and diabetes	
Variable types	N	β (95% CI)	P-value	β (95% CI)	P-value
Overall	300	0.462 (0.281-0.642)	<0.001	0.432 (0.247-0.618)	<0.001
Hemorrhage location					
Supratentorial	270	0.389 (0.203-0.576)	<0.001	0.334 (0.140-0.527)	0.001
Infratentorial	30	0.128 (-0.268-0.524)	0.512	N/A	N/A
Ventricular extension					
Yes	82	0.060 (0.011-0.108)	0.017	N/A	N/A
No	218	0.500 (0.294-0.705)	<0.001	0.016 (0.009-0.024)	<0.001
Surgery					
No	179	0.147 (-0.039-0.332)	0.121	N/A	N/A
Yes	121	0.430 (0.198-0.663)	<0.001	0.417 (0.184-0.650)	<0.001

β=regression coefficient; CI=confidence interval. The β values, 95% CIs, and p-values were obtained from the linear regression models. β values were interpreted as the change in mean ABC/2 scores (on the square root scale) for each fold of NfL concentration. NfL concentrations are shown on a base 2 logarithmic scale. The bleeding volume of intravascular hemolysis (IVH) was calculated using a 3D slicer. N/A, not available.

We next tested the associations between NfL and clinical scores, NIHSS, and GCS measured at the time of blood collection to evaluate the relationship between neurological deficit and coma severity. Analyses were conducted and stratified according to the hemorrhage location, ventricle extension, and surgical intervention. Overall, higher NfL was associated with higher NIHSS and GCS scores after adjusting for the time from stroke episode to blood collection (*P* < 0.001), as well as other potential confounding factors (*P* < 0.001), indicating an association with worse clinical manifestation (Table 3 and 4). In subgroup analyses, NfL was positively correlated with the NIHSS score of patients with supratentorial hemorrhage, those without ventricular extension, and those receiving surgical treatment (Table 3). However, the correlation did not exist for patients with infratentorial hemorrhage and patients without surgery and was present only nominally for patients with ventricular extension. Similar results were found for GCS scores (Table 4). Additionally, NfL concentrations on days 7 and 14 post-ICH were not associated with NIHSS and GCS scores (Supplemntary Table 1).

### Plasma NfL correlates with long-term outcomes of patients with intracerebral hemorrhage

Sixty-nine patients died during the follow-up, and the modified Rankin Scale (mRS) of the remaining 231 patients was repeatedly evaluated at 1, 3, 6, and 12 months after ICH, which allowed us to examine the relationship between NfL and long-term outcome of ICH. Higher NfL levels within 72 h were associated with an mRS ≥ 3 at 1, 6, and 12 months after adjusting for the time from hemorrhage to blood collection (*P* = 0.036, 0.044, 0.001, respectively). In further adjustments for potential confounding factors, such as age, sex, previous cerebral vascular disease (CVD), hypertension, diabetes, and smoking, the association remained significant only at 6 months (*P* = 0.002) and at 12 months (*P* < 0.001). We also examined whether the association between elevated NfL and higher mRS was independent of NIHSS and hemorrhage volume, which were additionally adjusted for NIHSS at blood collection, ABC/2, or both. The association between elevated NfL within 72 h and mRS at 6 or 12 months remained significant in further adjustments to NIHSS (*P* = 0.011 for 6 months, P = 0.003 for 12 months), ABC/2 (*P* = 0.038 at 6 months, *P* = 0.007 at 12 months), or both (*P* = 0.029 at 6 months, *P* = 0.005 at 12 months) (Table 5). We also examined the correlation between plasma NfL and mRS ≥ 3 on days 7 and 14 post-ICH, which were no longer significantly associated (Supplemtary Table 2).

To further evaluate the ability of NfL levels within 72 h to predict an mRS ≥ 3 at 6- and 12-months post-stroke, we estimated the area under the ROC curve (AUC) with and without NfL included in the multivariable logistic regression model that included NIHSS and ABC/2 at the time of blood collection. The inclusion of NfL in the model increased the AUC from 0.835 to 0.846 at 6 months and from 0.823 to 0.834 at 12 months (Supplemtary Table 3).

**Table 3:** Association of NfL concentrations with NIHSS scores at blood collection.

		Adjusting for the time from hemorrhagic to blood collection		Adjusting for the time from hemorrhagic to blood collection, age at blood collection, sex, current smoking, hypertension, cerebrovascular disease, and diabetes	
Variable types	N	β (95% CI)	P-value	β (95% CI)	P-value
Overall	300	0.295 (0.166-0.424)	<0.001	0.279 (0.140-0.418)	<0.001
Hemorrhage location					
Supratentorial	270	0.248 (0.132-0.364)	<0.001	0.239 (0.116-0.362)	<0.001
Infratentorial	30	-0.202 (-0.855-0.451)	0.529	N/A	N/A
Ventricular extension					
Yes	82	0.259 (-0.072-0.590)	0.123	N/A	N/A
No	218	0.310 (0.173-0.447)	<0.001	0.312 (0.158-0.466)	<0.001
Surgery					
No	179	0.053 (-0.102-0.209)	0.501	N/A	N/A
Yes	121	0.329 (0.140-0.518)	<0.001	0.318 (0.122-0.513)	<0.001

β=regression coefficient; CI=confidence interval. The β values, 95% CIs, and p-values were obtained from the linear regression models. β values were interpreted as the change in mean NIHSS scores (on the square root scale) for each fold of NfL concentration. NfL concentrations are shown on a base 2 logarithmic scale. N/A= not available; NIHSS= National Institutes of Health Stroke Scale.

### Plasma NfL predicts the survival of patients with intracerebral hemorrhage

Next, we investigated whether plasma NfL levels could predict the survival of patients with ICH. The date of blood collection was set as the baseline for survival analysis, and censoring was performed on the date of the last follow-up or death. During the follow-up period, 69 (23%) patients died. Higher NfL within 72 h was significantly associated with poorer survival for all patients with ICH [1.003, odds ratio (OR) (95% CI), 1.002-1.004; *P* < 0.001] after adjusting for the time from ICH to blood collection. These results remained consistent after further adjusting for age, sex, current smoking, hypertension, cerebrovascular disease history, and diabetes (1.857, 95%CI, 1.568-2.198, *P* < 0.001). We subsequently examined whether the association between NfL and survival was independent of hemorrhage volume or NIHSS score at the time of blood collection. In the multivariable model, after further adjustment, the association between NfL and survival remained significant [1.395; 95%CI, 1.150-1.692; *P* = 0.001] (Supplementary Table 4).

**Figure 2.**
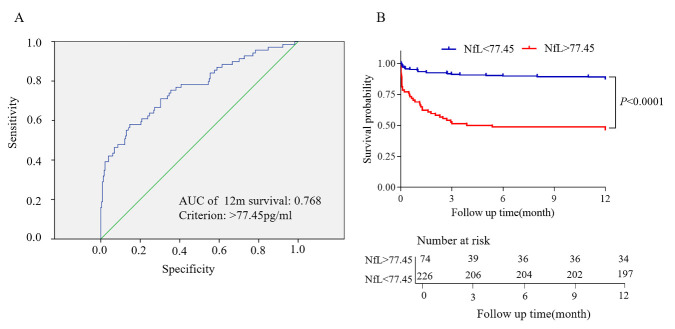
NfL concentrations measured within 72 hours of disease onset and survival within the 12-month interval. The association between NfL concentrations measured within 72 h of disease onset and survival within the 12-month interval was evaluated using Cox proportional hazard regression models. (A) The receiver operating characteristic (ROC) curve to assess the diagnostic accuracy of NfL in discriminating survival or mortality outcomes, area under the ROC (AUC), and criterion value are shown. AUC = 0.768; patients died (n = 69 (23%)). (B) Kaplan-Meier curve with a log-rank test of the predictor above and below the cut-off value. (NfL=77.45 pg/ml).

The area under the ROC curve in the multivariable model was calculated to examine the ability of NfL to predict survival at 12 months post-stroke independently. NfL levels differentiated non-survivors from survivors with an AUC of 0.768 at 12 months, and the optimal cut-off point was 77.45 pg/ml NfL. We also analyzed the Kaplan-Meier curve with the log-rank test of the predictor above and below the cut-off point, which revealed that the plasma NfL level above 77.45 pg/ml at admission increased the risk of death (*P*<0.001, Fig. 2).

### Plasma NfL correlates white matter injury and cognitive function at 6 months after ICH

**Figure 3.**
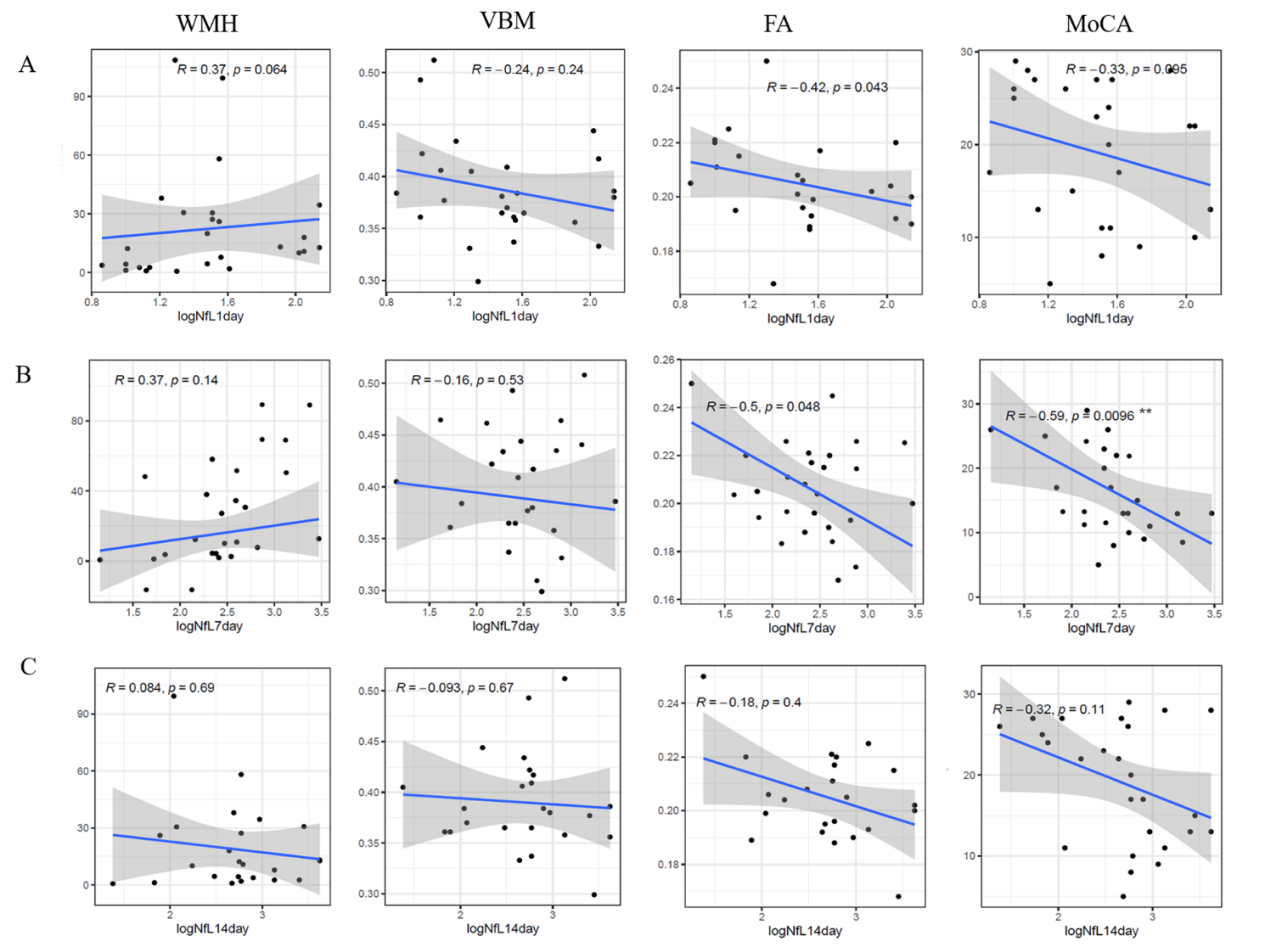
Correlation of plasma NfL at days 1, 7, and 14 with image markers and cognitive function at 6 months in ICH patients. Twenty-six patients were available for MRI and cognitive evaluation six months post-ICH. Plasma NfL concentrations on days 1, 7, and 14 after ICH were collected from these patients. The associations between NfL concentrations with imaging markers, cognitive function was evaluated using Spearman’s test. (A) Correlation between plasma NfL on 1 day and FA, WBM, WMH, and MoCA. (B) the correlation between plasma NfL on day 7 with FA, WBM, WMH, and MoCA. (C) Correlation between plasma NfL at 14 days with FA, WBM, WMH, and MoCA. NfL, neurofilament light chain; ICH, intracerebral hemorrhage; MoCA, Montreal cognitive assessment; WMH, white matter hyperintensities; VBM, voxel-based morphometry; FA, fractional anisotropy; n=26, Spearman’s rank correlation test, R and P values are shown in the figure.

To determine the relationship between plasma NfL and chronic brain tissue damage after ICH, we performed MRI scans in 26 patients at 6 months post-ICH and evaluated their cognitive function using the Montreal Cognitive Assessment (MoCA). The associations between quantified plasma NfL levels within 24 hours post-stroke, 7 days, and 14 days after ICH, and MRI markers including (i) white matter hyperintensities (WMH) volume; (ii) structural integrity of white matter fibers evaluated by FA value; (iii) gray matter/white matter density or volume measured by voxel-based morphometry (VBM), and cognitive function, were analyzed (Fig. 3). Plasma NfL showed a negative correlation with FA value, with the greatest statistical power identified for NfL levels at day 7 post-ICH (*r* = -0.5, *P* = 0.048), while no such correlation was detected for WMH and VBM at any of the collection time points. Additionally, plasma NfL was negatively correlated with MoCA, measurement of cognitive behavior, at 6 months post-ICH, and this correlation was most significant at day 7 NfL concentrations (*r* = -0.59, *P* < 0.01). These results indicate that plasma NfL, especially in the subacute phase, is closely associated with white matter fiber integrity and cognitive function six months after ICH.

**Table 4:** Association of NfL concentrations with GCS scores at blood collection.

		Adjusting for the time from hemorrhagic to blood collection		Adjusting for the time from hemorrhagic to blood collection, age at blood collection, sex, current smoking, hypertension, cerebrovascular disease, and diabetes	
Variable types	N	β (95% CI)	P-value	β (95% CI)	P-value
Overall	300	-0.077 (-0.115 - -0.040)	<0.001	-0.086 (-0.125 - -0.048)	<0.001
Hemorrhage location					
Supratentorial	270	-0.088 (-0.124 - -0.053)	<0.001	-0.088 (-0.125 - -0.050)	<0.001
Subtentorial	30	-0.050 (-0.256 - 0.156)	0.620	N/A	N/A
Ventricular extension					
Yes	82	-0.078 (-0.159 - 0.003)	0.059	N/A	N/A
No	218	-0.077 (-0.118 - -0.037)	<0.001	-0.081 (-0.125 - -0.037)	<0.001
Surgery					
No	179	-0.060 (-0.110 - -0.011)	0.017	-0.066 (-0.117 - -0.015)	0.011
Yes	121	-0.016 (-0.116 - -0.006)	0.030	-0.073 (-0.129 - -0.016)	0.012

β =regression coefficient; CI=confidence interval. The β values, 95% CIs, and p-values were obtained from the linear regression models. β values were interpreted as the change in mean GCS scores (on the base-2 logarithmic scale) for each fold of NfL concentration. NfL concentrations are shown on a base 2 logarithmic scale. N/A = not available; GCS=Glasgow score.

## DISCUSSION

Patients with ICH often suffer from heterogeneous and unpredictable long-term deficiencies, such as cognitive decline, which is independent of the primary characteristics of hemorrhage. However, the ability to accurately predict and manage these outcomes for ICH survivors remains far from optimal due to the lack of a reliable biomarker. As a widely expressed neuronal protein, NfL has been identified as a biomarker of axonal injury in nearly all neurodegenerative disorders and vascular conditions [16, 25, 28]. Here, we report the plasma NfL levels measured at the acute and subacute time points following a hemorrhagic stroke in 300 ICH patients with long-term outcome measures available. We observed a significant elevation in plasma NfL levels in patients with ICH compared to healthy controls. Notably, circulating NfL concentrations increased after ICH in a bimodal trend; a primary increase was detected within 24 h of ictus, followed by a secondary increase within two weeks thereafter. Plasma NfL levels within 72 h were closely associated with hematoma volume and disease severity and were predictive of long-term functional outcomes and mortality in patients with ICH. Additionally, plasma NfL, especially detected on day 7, was correlated with white matter integrity and cognitive function 6 months after ICH. These data provide compelling insights into the NfL profile post-ICH and advance the investigation of its suitability as a valuable marker to monitor acute brain injury with prognostic value for long-term outcomes.

**Table 5:** Association of NfL within 72 hours with modified Rankin scale at different follow-up times in ICH patients.

	Adjustment for the time from hemorrhagic to blood collection		Adjustment for the time from hemorrhagic to blood collection, age, sex, current smoking, hypertension, cerebrovascular disease history, and diabetes		Additional adjustment of initial multivariable model^1^ for NIHSS at blood collection		Additional adjustment of initial multivariable model^1^ for ABC/2 at blood collection		Additional adjustment of initial multivariable model^1^ for NIHSS and ABC/2 at blood collection	
Follow-up time	OR (95% CI)	P value	OR (95% CI)	P value	OR (95% CI)	P value	OR (95% CI)	P value	OR (95% CI)	P value
1 month	1.294 (1.017-1.646)	0.036	1.235 (0.957-1.593)	0.104	1.279 (0.949-1.723)	0.105	1.177 (0.902-1.537)	0.230	1.233 (0.908-1.675)	0.179
3 months	1.244 (1.006-1.538)	0.044	1.203 (0.962-1.506)	0.105	1.161 (0.919-1.468)	0.211	1.114 (0.875-1.417)	0.381	1.103 (0.861-1.413)	0.436
6 months	1.462 (1.157-1.849)	0.001	1.485 (1.152-1.915)	0.002	1.413 (1.083-1.845)	0.011	1.370 (1.032,1.819)	0.029	1.359 (1.018-1.814)	0.038
12 months	1.693 (1.285-2.231)	< 0.001	1.792 (1.304-2.463)	0.000	1.684 (1.201-2.361)	0.003	1.671 (1.165-2.397)	0.005	1.675 (1.154-2.431)	0.007

CI=confidence interval. Odds ratios, 95% CIs, and p-values resulting from binary logistic regression models. ORs were interpreted as multiplicative increases in the odds of a modified Rankin Scale score ≥ 3 for each fold increase in NfL concentrations. The complete multivariable analysis was adjusted for the time from stroke to blood collection, age, sex, current smoking, hypertension, cerebrovascular disease, and diabetes. The bleeding volume of intravascular hemolysis (IVH) was calculated using a 3D slicer. mRS, modified Rankin Scale; NIHSS, National Institutes of Health Stroke Scale; GCS, Glasgow Coma Score.

Blood NfL levels were often detected in the acute phase of various diseases in previous studies of ischemic stroke [25, 29], where NfL detection on day 3 was considered representative of the acute timeframe. Studies reporting NfL changes after ischemic stroke are limited by this timeframe. A probable reason for these time parameters could be based on the assumption that NfL release coincides with stroke primary injury, which occurs within minutes to hours post-ictus. Consequently, the temporal dynamics of NfL after an acute stroke have not been thoroughly described in previous studies. Here, we observed that after ICH, plasma NfL rapidly increased at admission and peaked at approximately 24 h. NfL detected in plasma then declined to a relatively lower level until day 3 post-stroke. However, the continued detection of NfL on days 7 and 14 in a subset of patients identified a subsequent increase in plasma NfL on day 14 after ICH, suggesting a secondary and consecutive neuroaxonal injury after ICH. Thus, plasma NfL helped sensitively trace neuroaxonal injury post-ICH, suggesting that the disease course after ICH can be segmented into the first 24 h as an acute injury phase and a secondary injury phase thereafter.

Plasma NfL within 72 h is closely associated with hemorrhage volume and clinical manifestations at admission; however, this correlation was not extended for NfL detected on days 7 and 14. This suggests that disparate pathogenic processes may contribute to axonal injury during the two stages of ICH. Furthermore, our analyses revealed that NfL detected at the later stage is associated with white matter damage as well as a decline in cognitive performance in patients 6 months after ICH, suggesting that plasma NfL levels in the subacute stage may be a potential predictor of long-term cognitive decline in ICH patients, which are reportedly independent of acute hemorrhage characteristics.

We found that long-term functional ability and survival were independently associated with NfL levels within 72 h and demonstrated that NfL is predictive of long-term functional capacity and survival of patients within 12 months after ICH. This finding strongly supports the potential use of plasma NfL levels in the acute phase after ICH as a biomarker to assist in the long-term prognostication of patients with ICH. Nonetheless, owing to the complexity of the pathophysiological features of chronic ICH and diversified long-term outcomes, incorporating other potential markers together with NfL could be a more reasonable choice for future practice and a strategy worth pursuing.

Due to different study designs and measurement methods, it is challenging to directly compare the capacity of NfL against other biomarkers, such as hematoma volume and ICH score, in predicting the long-term outcomes of ICH patients. However, as a neuron-specific biomarker, NfL directly reflects the extent of neuroaxonal injury of the brain and closely correlates with the imaging characteristics of ICH. Given its easy sampling and detection, plasma NfL can be adapted to measure neuronal injury in real time. Phosphorylated neurofilament heavy chain (pNfH), a neuron-specific biomarker, is reportedly an independent predictor of 6-month mortality and unfavorable outcome (mRS > 2) [30], with similar specificity and sensitivity to NfL, compared to both reported [26] and present studies. Direct comparison of the predictive capacity of pNfH and NfL is thus warranted in future studies, and whether combined measurement of both could increase the predictive value is also worth determining.

The associations between plasma NfL concentrations and clinical outcomes of ICH patients have been reported previously in two studies [25, 26]. One study recruited 37 spontaneous ICH patients and showed that plasma NfL levels within 24 h of ictus are associated with 30-day mortality [26]. Another study [25], which retrospectively studied the associations between blood NfL and disease outcome in a mixed stroke population, including acute cerebral infarction (n = 227), aneurysmal subarachnoid hemorrhage (n = 58), and ICH (n = 29), concluded that blood NfL levels are associated with acute stroke severity, 3- or 6- month functional disability, and mortality. The conclusions were further validated in two independent ICH cohorts comprising 96 and 54 patients. Compared to those two studies, the present study is the largest study investigating blood NfL levels in patients with ICH. Additionally, our study provided several new insights into the field, which include the correlation between plasma NfL and long-term outcomes up to 12 months post-ICH, dynamic change of NfL in plasma from acute to subacute stages of ICH, and the association of NfL with brain structure changes on MRI at the chronic phase. These new insights encourage future studies to uncover the changes in plasma NfL in the chronic stage and explore their relationship with late-onset comorbidities, such as cognitive decline, in the survival of ICH patients.

Our study had several limitations. First, hospital-based recruitment from a specific geographical area may limit the generalizability of our results. Second, due to the nature of the study, most patients were unreachable for on-site follow-up and repeated measurement of NfL levels at later time points. Furthermore, MRI measurements were only available for a small portion of the study population, limiting our ability to examine specific associations and late-onset changes in patients with ICH. Nevertheless, our results provide relatively robust evidence for the potential value of plasma NfL within 72 h of disease onset as a biomarker for predicting the long-term outcomes of spontaneous ICH.

Our data suggest that plasma NfL is reliably associated with long-term outcomes and survival in patients with ICH. Upon establishing normative NfL values according to age and other relevant parameters in healthy Chinese individuals [27], we expect NfL detection to be implemented soon in the clinics as a substitute or corollary for repeated head imaging and as a supplement to hematoma volume and stroke severity assessment for predicting the prognosis of ICH.

## Supplementary Materials

The Supplementary data can be found online at: http://www.aginganddisease.org/EN/10.14336/AD.2022.21020.

## References

[1] An SJ, Kim TJ, Yoon BW (2017). Epidemiology, risk factors, and clinical features of intracerebral hemorrhage: An update. J Stroke, 19:3-10. 28178408 10.5853/jos.2016.00864 PMC5307940

[2] van Asch CJ, Luitse MJ, Rinkel GJ, van der Tweel I, Algra A, Klijn CJ (2010). Incidence, case fatality, and functional outcome of intracerebral haemorrhage over time, according to age, sex, and ethnic origin: A systematic review and meta-analysis. Lancet Neurol, 9:167-176. 20056489 10.1016/S1474-4422(09)70340-0

[3] Lu C, Tan C, Ouyang H, Chen Z, Yan Z, Zhang M (2022). Ferroptosis in Intracerebral Hemorrhage: A Panoramic Perspective of the Metabolism, Mechanism and Theranostics. Aging Dis, 13:1348-1364. 36186133 10.14336/AD.2022.01302 PMC9466971

[4] Hemphill JC 3rd, Greenberg SM, Anderson CS, Becker K, Bendok BR, Cushman M, et al. (2015). Guidelines for the management of spontaneous intracerebral hemorrhage: A guideline for healthcare professionals from the American Heart Association/American Stroke Association. Stroke, 46:2032-2060. 26022637 10.1161/STR.0000000000000069

[5] Biffi A, Bailey D, Anderson CD, Ayres AM, Gurol EM, Greenberg SM, et al. (2016). Risk factors associated with early vs delayed dementia after intracerebral hemorrhage. JAMA Neurol, 73:969-976. 27295605 10.1001/jamaneurol.2016.0955 PMC5327781

[6] Xu M, Cheng Y, Song Q, Yuan R, Zhang S, Hao Z, et al. (2019). Total Burden of Cerebral small vessel disease in Recurrent ICH versus first-ever ICH. Aging Dis, 10:570-577. 31165001 10.14336/AD.2018.0804 PMC6538213

[7] Hemphill JC 3rd, Farrant M, Neill TA Jr, (2009). Prospective validation of the ICH Score for 12-month functional outcome. Neurology, 73:1088-1094. 19726752 10.1212/WNL.0b013e3181b8b332 PMC2764394

[8] Chen X, Jin Y, Chen J, Chen X, Cao X, Yu L, et al. (2018). Relationship between white matter hyperintensities and hematoma volume in patients with intracerebral hematoma. Aging Dis, 9:999-1009. 30574413 10.14336/AD.2018.0108 PMC6284763

[9] Schwarz G, Kanber B, Prados F, Browning S, Simister R, Jäger R, et al. (2022). Acute corticospinal tract diffusion tensor imaging predicts 6-month functional outcome after intracerebral haemorrhage. J Neurol, 269:6058-6066. 35861854 10.1007/s00415-022-11245-1 PMC9553831

[10] Urday S, Kimberly WT, Beslow LA, Vortmeyer AO, Selim MH, Rosand J, et al. (2015). Targeting secondary injury in intracerebral haemorrhage--Perihaematomal oedema. Nat Rev Neurol, 11:111-122. 25623787 10.1038/nrneurol.2014.264

[11] Leasure AC, Kuohn LR, Vanent KN, Bevers MB, Kimberly WT, Steiner T, et al. (2021). Association of serum IL-6 (interleukin 6) with functional outcome after intracerebral hemorrhage. Stroke, 52:1733-1740. 33682454 10.1161/STROKEAHA.120.032888 PMC8085132

[12] Wang Z, Wu X, Yan T, Liu M, Yu W, Du Q, et al. (2022). Elevated plasma complement C1q levels contribute to a poor prognosis after acute primary intracerebral hemorrhage: A prospective cohort study. Front Immunol, 13:920754. 35812425 10.3389/fimmu.2022.920754 PMC9259799

[13] Li S, Wang Y, Wang W, Zhang Q, Wang A, Zhao X (2022). Stress hyperglycemia is predictive of clinical outcomes in patients with spontaneous intracerebral hemorrhage. BMC Neurol, 22:236. 35761206 10.1186/s12883-022-02760-9 PMC9235136

[14] Rindler RS, Allen JW, Barrow JW, Pradilla G, Barrow DL (2020). Neuroimaging of intracerebral hemorrhage. Neurosurgery, 86:E414-E423. 32109294 10.1093/neuros/nyaa029

[15] Scherling CS, Hall T, Berisha F, Klepac K, Karydas A, Coppola G, et al. (2014). Cerebrospinal fluid neurofilament concentration reflects disease severity in frontotemporal degeneration. Ann Neurol, 75:116-126. 24242746 10.1002/ana.24052 PMC4020786

[16] Khalil M, Teunissen CE, Otto M, Piehl F, Sormani MP, Gattringer T, et al. (2018). Neurofilaments as biomarkers in neurological disorders. Nat Rev Neurol, 14:577-589. 30171200 10.1038/s41582-018-0058-z

[17] Barro C, Benkert P, Disanto G, Tsagkas C, Amann M, Naegelin Y, et al. (2018). Serum neurofilament as a predictor of disease worsening and brain and spinal cord atrophy in multiple sclerosis. Brain, 141:2382-2391. 29860296 10.1093/brain/awy154

[18] Lin YS, Lee WJ, Wang SJ, Fuh JL (2018). Levels of plasma neurofilament light chain and cognitive function in patients with Alzheimer or Parkinson disease. Sci Rep, 8:17368. 30478269 10.1038/s41598-018-35766-w PMC6255914

[19] Uphaus T, Bittner S, Grschel S, Steffen F, Muthuraman M, Wasser K, et al. (2019). NfL (Neurofilament Light Chain) Levels as a Predictive Marker for Long-Term Outcome After Ischemic Stroke. Stroke, 50:3077-3084. 31537188 10.1161/STROKEAHA.119.026410

[20] Cheng X, Su Y, Wang Q, Gao F, Ye X, Wang Y, et al. (2020). Neurofilament light chain predicts risk of recurrence in cerebral amyloid angiopathy-related intracerebral hemorrhage. Aging (Albany, NY), 12:23727-23738. 33221749 10.18632/aging.103927 PMC7762473

[21] Hviid CVB, Lauridsen SV, Gyldenholm T, Sunde N, Parkner T, Hvas AM (2020). Plasma neurofilament light chain is associated with poor functional outcome and mortality rate after spontaneous subarachnoid hemorrhage. Transl Stroke Res, 11:671-677. 31808039 10.1007/s12975-019-00761-4

[22] Garland P, Morton M, Zolnourian A, Durnford A, Gaastra B, Toombs J, et al. (2021). Neurofilament light predicts neurological outcome after subarachnoid haemorrhage. Brain, 144:761-768. 33517369 10.1093/brain/awaa451 PMC8041040

[23] Graham NSN, Zimmerman KA, Moro F, Heslegrave A, Maillard SA, Bernini A, et al. (2021). Axonal marker neurofilament light predicts long-term outcomes and progressive neurodegeneration after traumatic brain injury. Sci Transl Med, 13:eabg9922. 34586833 10.1126/scitranslmed.abg9922

[24] Muraoka S, DeLeo AM, Yang Z, Tatebe H, Yukawa-Takamatsu K, Ikezu S, et al. (2021). Proteomic profiling of extracellular vesicles separated from plasma of former National Football League players at risk for chronic traumatic encephalopathy. Aging Dis, 12:1363-1375. 34527415 10.14336/AD.2020.0908 PMC8407879

[25] Gendron TF, Badi MK, Heckman MG, Jansen-West KR, Vilanilam GK, Johnson PW, et al. (2020). Plasma neurofilament light predicts mortality in patients with stroke. Sci Transl Med, 12. 10.1126/scitranslmed.aay1913 PMC9534269 33177179

[26] Hviid CVB, Gyldenholm T, Lauridsen SV, Hjort N, Hvas AM, Parkner T (2020). Plasma neurofilament light chain is associated with mortality after spontaneous intracerebral hemorrhage. Clin Chem Lab Med, 58:261-267. 31494627 10.1515/cclm-2019-0532

[27] Chen J, Yang X, Zhang Y, Zheng P, Wei C, Mao Z, et al. (2021). Reference values for plasma neurofilament light chain (NfL) in healthy Chinese. Clin Chem Lab Med, 59:e153-e156. 33068379 10.1515/cclm-2020-1030

[28] Benatar M, Zhang L, Wang L, Granit V, Statland J, Barohn R, et al. (2020). Validation of serum neurofilaments as prognostic and potential pharmacodynamic biomarkers for ALS. Neurology, 95:e59-e69. 32385188 10.1212/WNL.0000000000009559 PMC7371380

[29] Pedersen A, Stanne TM, Nilsson S, Klasson S, Rosengren L, Holmegaard L, et al. (2019). Circulating neurofilament light in ischemic stroke: Temporal profile and outcome prediction. J Neurol, 266:2796-2806. 31375988 10.1007/s00415-019-09477-9 PMC6803587

[30] Cai JY, Lu C, Chen MH, Ba HJ, Chen XD, Lin JH, et al. (2013). Predictive value of phosphorylated axonal neurofilament subunit H for clinical outcome in patients with acute intracerebral hemorrhage. Clin Chim Acta, 424:182-186. 23810564 10.1016/j.cca.2013.06.019

