# Supplementary material for: Plasma Neurofilament Light Chain Predicts Mortality and Long-Term Neurological Outcomes in Patients with Intracerebral Hemorrhage
Source: Aging Dis. 2023 Apr 1;14(2):560–71. doi: 10.14336/AD.2022.21020 (PMC10017162; doi:10.14336/AD.2022.21020)
Supplement: Supplementary file 1 — The Supplementary data can be found online at: http://www.aginganddisease.org/EN/10.14336/AD.2022.21020. [file AD-14-2-560-s.pdf]

SUPPLEMENTARY DATA

Plasma Neurofilament Light Chain Predicts Mortality and Long-Term Neurological Outcomes in Patients with Intracerebral Hemorrhage

Pei Zheng[1], Xuejiao Wang[2], Jingshan Chen[3], Xinli Wang[3], Samuel X Shi[4*], Kaibin Shi[1*]

© 2022. Zheng P et al. Published online at http://www.aginganddisease.org/EN/10.14336/AD.2022.21001

SUPPLEMENTARY DATA

Supplementary Table 1. Associations of NfL concentrations at Days 7 and 14 with ABC/2, NIHSS and GCS scores at blood collection.

	NfL concentrations day 7			NfL concentrations day 14		
	Univariable model			Univariable model		
**Variable types**	N	β (95% CI)	P value	N	β (95% CI)	P value
ABC/2	41	5.00 (-2.63, 12.63)	0.191	41	18.16 (2.89, 33.43)	0.021
NIHSS	41	18.39 (-23.82, 60.61)	0.381	41	16.52 (-45.51, 78.54)	0.594
GCS	41	5.07 (-91.70, 101.85)	0.915	41	34.31 (-141.13, 209.76)	0.695

β=regression coefficient; CI=confidence interval. β values, 95% CIs, and p-values result from linear regression models. β values are interpreted as the change in mean ABC/2, NIHSS and GCS scores for each doubling in NfL concentrations. NIHSS= National Institutes of Health Stroke Scale. GCS=Glasgow coma scale.

Supplementary Table 2. Associations of NfL concentrations at Days 7 and 14 with modified Rankin scale at different follow-up time in patients with ICH.

	NfL concentrations day 7			NfL concentrations day 14		
	N	Univariable model		N	Univariable model	
Follow-up time		OR (95% CI)	P value		OR (95% CI)	P value
1 month	41	1.014(1.002, 1.025)	0.021	41	1.005(1.001, 1.009)	0.025
3 months	41	1.004(0.999, 1.009)	0.098	41	1.002(1.000, 1.004)	0.040
6 months	41	1.001(0.999, 1.003)	0.321	41	1.000(1.000, 1.001)	0.687
12 months	41	1.000(0.997, 1.002)	0.715	41	1.000(0.999, 1.001)	0.865

OR=odds ratio; CI=confidence interval. ORs, 95% CIs, and p-values result from binary logistic regression models. ORs are interpreted as the multiplicative increase in the odds of a modified Rankin Scale score > 3 for each doubling in NfL concentrations. A rigorous multivariable analysis was not performed due to small sample size. mRS=modified Rankin Scale.

Supplementary Table 3. Examination of the ability of NfL concentrations to independently predict 6 and 12-month mRS scores.

		Full multivariable model with ABC/2 at hemorrhage[1]		Full multivariable model with NIHSS at blood collection[2]		Full multivariable model with ABC/2 and NIHSS at blood collection[3]	
**Follow-up time**	Predictive ability measure	Without NfL concentrations	With NfL concentrations	Without NfL concentrations	With NfL concentrations	Without NfL concentrations	With NfL concentrations
6 months	AUC	0.787	0.800	0.798	0.819	0.835	0.846
12 months	AUC	0.800	0.803	0.790	0.807	0.823	0.834

AUC=area under the ROC curve. AUCs result from binary logistic regression models. [1] This full multivariable was adjusted for time from ICH to blood collection, age at blood collection, sex, current smoking, hypertension, cerebrovascular disease, diabetes and ABC/2 score at hemorrhage. [2] This full multivariable was adjusted for time from ICH to blood collection, age at blood collection, sex, current smoking, hypertension, cerebrovascular disease, diabetes and NIHSS scores at blood collection. [3] This full multivariable analysis was adjusted for time from ICH to blood collection, age at blood collection, sex, current smoking, hypertension, cerebrovascular disease, diabetes, ABC/2 and NIHSS scores at blood collection.

SUPPLEMENTARY DATA

Supplementary Table 4. Associations between NfL concentrations within 72 hours and survival after ICH.

	Adjusting for time from ICH to blood collection		Adjusting for time from ICH to blood collection, age, sex, current smoking, hypertension, cerebrovascular disease history and diabetes		Additionally adjusting the initial multivariable model* for NIHSS at blood collection		Additionally adjusting the initial multivariable model* for ABC/2		Additionally adjusting the initial multivariable model* for NIHSS and ABC/2 at blood collection	
**Variables**	**HR (95% CI)**	**P value**	**HR (95% CI)**	**P value**	**HR (95% CI)**	**P value**	**HR (95% CI)**	**P value**	**HR (95% CI)**	**P value**
mortality	1.003 (1.002, 1.004)	<0.001	1.857 (1.568, 2.198)	<0.001	1.443 (0.208, 1.723)	<0.001	1.609 (1.332, 1.944)	<0.001	1.395 (1.150, 1.692)	0.001

Hazard ratios result from Cox proportional hazards regression models. *The initial multivariable was adjusted for time from ICH to blood collection, age at blood collection, sex, current smoking, hypertension, cerebrovascular disease and diabetes. HR= Hazard ratio.

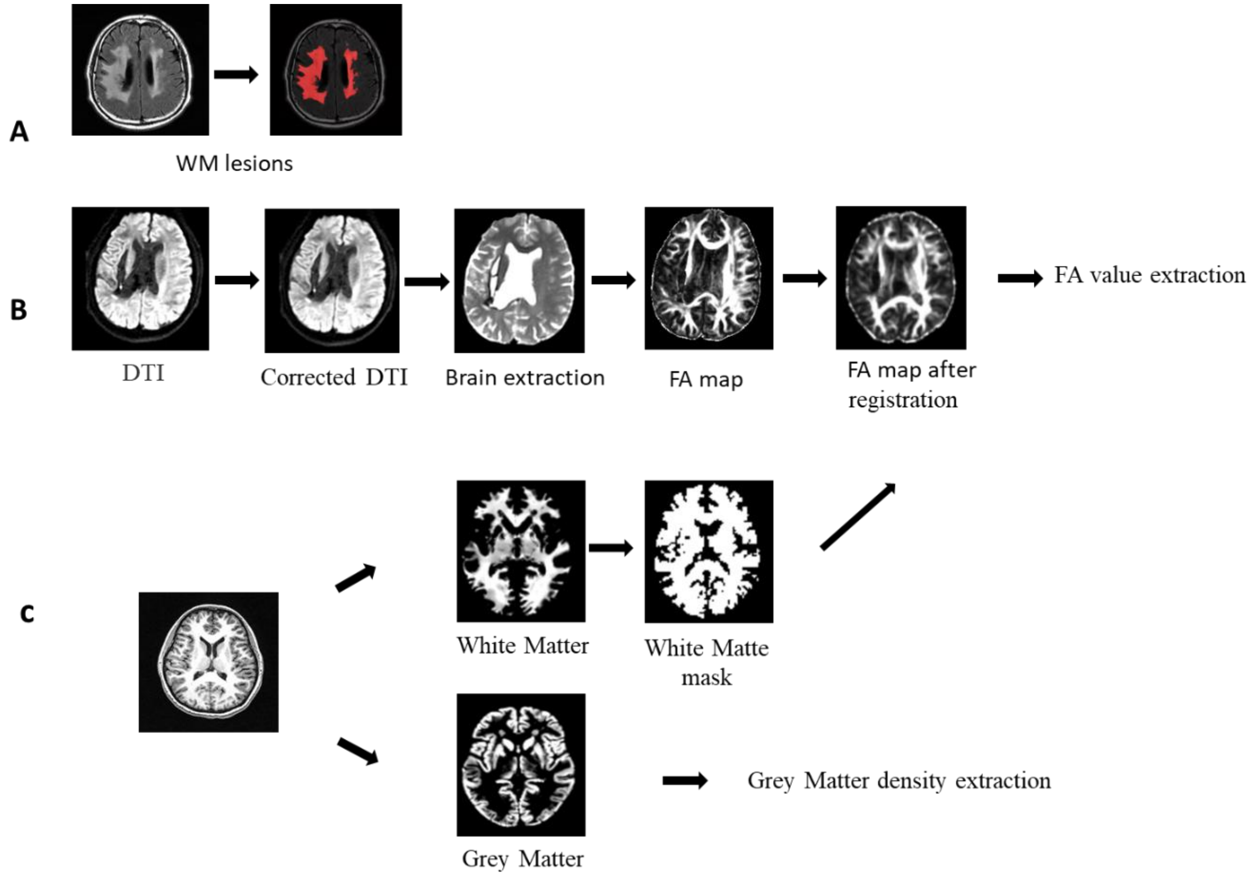

Supplementary Figure 1. MRI data analysis pipeline. MRI imaging data including T2-Weighted fluid-attenuated inversion recovery (FLAIR), diffusion tensor imaging (DTI) and T1 structural images were processed using the following approaches. (A) WM lesions were labeled from T2- FLAIR images using 3D-slicer. (B) DTI images are eddy-corrected, undergo brain extraction, FSL DTIFIT extraction of tensors and FLIRT linear registration. The normalized, modulated, and smoothed WM density maps were used as a mask to extract the FA values of these patients for statistical analysis. (C) T1 structural images are segmented in CAT12 Toolbox producing grey matter, white matter and CSF maps. GM density maps were used for the statistical analysis.

© 2022. Zheng P et al. Published online at http://www.aginganddisease.org/EN/10.14336/AD.2022.21001

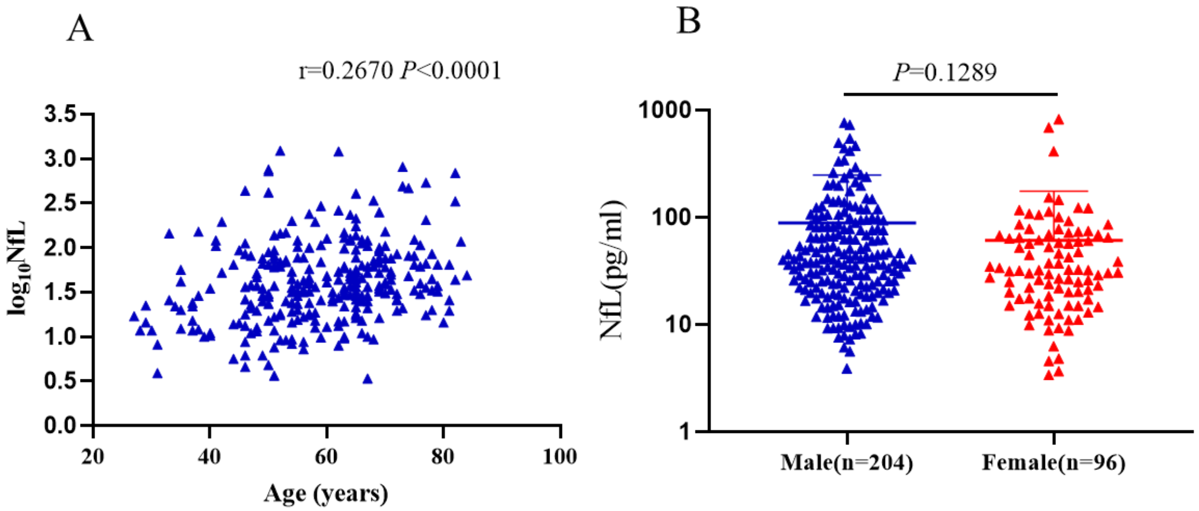

Supplementary Figure 2. Correlation of plasma NfL with age or sex in patients with ICH. (A) The linear regression plot shows the correlation between plasma NfL and age in patients with ICH. (B) Interleaved scatter graph shows plasma NfL concentrations of the male and female patients with ICH. Data are presented as the median with interquartile values. The Y axis of NfL concentrations is shown on the base 10 logarithmic scale. Linear regression was performed in A and Mann-Whitney U test in B, p values are shown in the figure.

© 2022. Zheng P et al. Published online at http://www.aginganddisease.org/EN/10.14336/AD.2022.21001
